# Dynamics and Collapse in a Power System Model with Voltage Variation: The Damping Effect

**DOI:** 10.1371/journal.pone.0165943

**Published:** 2016-11-10

**Authors:** Jinpeng Ma, Yong Sun, Xiaoming Yuan, Jürgen Kurths, Meng Zhan

**Affiliations:** 1 Wuhan Institute of Physics and Mathematics, Chinese Academy of Sciences, Wuhan, China; 2 University of the Chinese Academy of Sciences, Beijing, China; 3 State Key Laboratory of Advanced Electromagnetic Engineering and Technology, School of Electrical and Electronic Engineering, Huazhong University of Science and Technology, Wuhan, China; 4 Potsdam Institute for Climate Impact Research (PIK), Potsdam, Germany; 5 Department of Physics, Humboldt University, Berlin, Germany; 6 Institute for Complex Systems and Mathematical Biology, University of Aberdeen, Aberdeen, United Kingdom; Universidad Rey Juan Carlos, SPAIN

## Abstract

Complex nonlinear phenomena are investigated in a basic power system model of the single-machine-infinite-bus (SMIB) with a synchronous generator modeled by a classical third-order differential equation including both angle dynamics and voltage dynamics, the so-called flux decay equation. In contrast, for the second-order differential equation considering the angle dynamics only, it is the classical swing equation. Similarities and differences of the dynamics generated between the third-order model and the second-order one are studied. We mainly find that, for positive damping, these two models show quite similar behavior, namely, stable fixed point, stable limit cycle, and their coexistence for different parameters. However, for negative damping, the second-order system can only collapse, whereas for the third-order model, more complicated behavior may happen, such as stable fixed point, limit cycle, quasi-periodicity, and chaos. Interesting partial collapse phenomena for angle instability only and not for voltage instability are also found here, including collapse from quasi-periodicity and from chaos etc. These findings not only provide a basic physical picture for power system dynamics in the third-order model incorporating voltage dynamics, but also enable us a deeper understanding of the complex dynamical behavior and even leading to a design of oscillation damping in electric power systems.

## Introduction

Electric power system is a very complicated dynamical system [1], due to its intrinsic property of high nonlinearity. Its dynamical behavior is of essential importance for a secure and stable operation of power systems, such as rotor-angle stability, voltage instability (or voltage collapse), and frequency stability. Therefore, it is very natural that the study of dynamical behavior in power systems, namely, the so-called power system dynamics [2], has been a very important topic in power electrical engineering. Meanwhile, it is no doubt that nonlinear dynamics, which has been developed to study general dynamical systems with nonlinearity, has become an inevitable and important mathematical tool in power system dynamics.

In most cases, the electromechanical dynamics of a synchronous generator is extremely important for that of the whole power system, as it generates and exports power to grids and thus it is generally believed as the source of the system. Its dynamics is dominated by the rotor motion, which can be represented by a well-known second-order differential equation in the simplest form, called the *swing equation* [3–12]. The explicit form of equation will be given subsequently. The swing equation plays a central role in characterizing many dynamical behaviors in power systems, such as rotor-angle stability in the simplest power system with one synchronous generator connected to an infinitely large node, namely, the single-machine-infinite-bus (SMIB) power system, and also in coupled multiple machines system. Very interestingly, in mathematics it is exactly the same as the dynamic equations in many other systems, such as forced pendulum in mechanics and the classical mechanistic description of superconducting Josephson junctions in physics [13]. For different parameters, three distinct dynamic regimes including stable fixed point (also called equilibrium state or equilibrium point), stable limit cycle, and their coexistence for different initial conditions, have been well recognized. Correspondingly, their critical bifurcation parameters, bistability, and hysteresis have been intensively studied and summarized in the standard textbook of nonlinear dynamics [13]. Recently, some researchers, however, have found that the dominant regime for a stable limit cycle is even larger than what previously researchers thought [14].

In studying power system dynamics, many researchers have further investigated more complicated dynamical behaviors with new models, such as considering the second-order SMIB power system under a periodic load disturbance [15–18], and found that a chaotic regime is very common. The SMIB system under Gaussian white noise excitation has been found showing induced and enhanced chaos and basin erosion [19, 20]. In addition, the second-order swing model can be extended to a third-order one which also considers the transient voltage, namely, the so-called *flux decay equation* [21–25]. Based on the nonlinear excitation control, its transient stability was analyzed. Additionally, it was proved that after the subcritical Hopf bifurcations the excitation control with a voltage control device could induce sustained oscillations and the hard-limit can induce a stable limit cycle and further chaotic motions via a sequence of period-doubling bifurcations [26, 27]. Researchers even found the rare phenomenon of the coexistence of four different attractors including a stable equilibrium point, a stable limit cycle and two strange attractors [28]. A forth-order time-delayed model of power system was studied and coexisting phenomenon was discovered extensively [29]. Basically, most of previous work mainly discussed a proper design of nonlinear excitation controller, but a detailed analysis of the nonlinear dynamic behaviors in the third-order power system is missing. Historically there were many detailed models, which were mathematically not tractable and could not be simulated under previous computational conditions, and the swing equations emerged as a reduced model under many assumptions. (See, e.g., the references [7, 8] or the recent survey [9].)

The purpose of this contribution is just to analyze the nonlinear dynamic behaviors of the third-order flux decay system. It has been well-known that the damping coefficient is related to the state variables of the power system, and the damping coefficient may be positive or negative [30]. In [31] a nonlinear damping model (empirical formula) was proposed with damping consisting of two components including one positive damping (rotor-damping) and the other negative damping (stator-damping). It is interesting to study the influence of the damping effect on the system’s dynamics, especially the negative one, which has not been investigated in all previous studies, to the best of our knowledge. In particular, we find several novel types of system collapses for phase dynamics only. A theoretical analysis based on the local stability of equilibrium states has been conducted, showing a perfect fit with the numerical results. These findings are of potential use for several relevant problems in power systems, such as voltage stability, voltage collapse, the relations between voltage stability and rotor-angle stability, oscillation and chaotic behaviors, etc.

The remainder of this paper is organized as follows: In the section of mathematical model, the third-order SMIB model of a power system is introduced. The results for its dynamic behaviors by considering both positive and negative damping coefficients are presented in detail in the section of rich dynamics and system collapse. In the section of theoretic analysis, the local stability of the equilibrium state is studied. Finally, the conclusions and discussions are given.

## Mathematical Model

**Fig 1:**
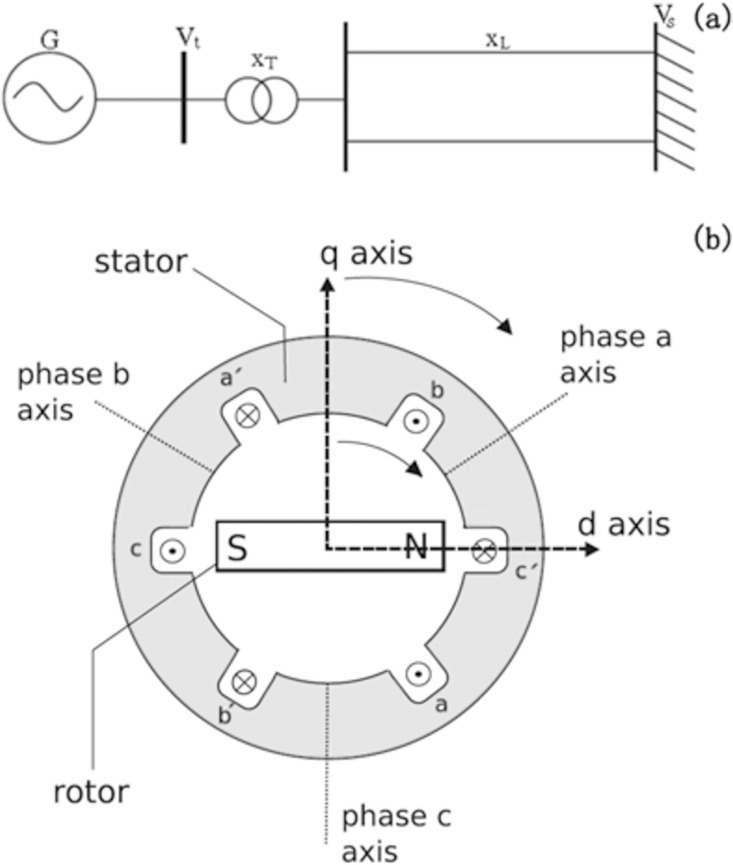
**(a) and (b) Schematic shows for the single-machine-infinite-bus power system and the coordinate systems in a synchronous generator, respectively.** In (a), here *G* represents the synchronous generator, *x*_*T*_ indicates the reactance of the transformer, *x*_*L*_ is the reactance of the transmission line, *V*_*s*_ denotes the infinite bus voltage, and *V*_*t*_ denotes the terminal voltage of the synchronous generator. In (b), the fixed abc coordinate system is defined by the aa', bb' and cc' axis of the stator with the dq coordinate system rotating with the rotor (d-axis centered in the rotor field's magnetic north pole N and the q-axis perpendicular to the S-N axis).

Fig 1(A) schematically shows the SMIB power system, where the electric power is generated from the synchronous generator (*G*) and transferred to an infinitely large bus through the transformer (*X*_*T*_) and two parallel transmission lines (*X*_*L*_). Here the infinite bus means that the voltage magnitude and phase are constant and unchanged with time. Due to its simplicity, this representation has been extensively used in the study of power system dynamics. The models of power systems can be presented at several diverse levels of complexity, relying on the studied problems. (See our discussions in the introduction part.) Here with some typical assumptions, we will employ and derive the classical third-order flux decay model describing also the voltage dynamics [32–34].

According to the basic principle of a synchronous generator, its rotor motion equation can be represented by

$$\dot{\delta }=\omega$$ (1)

$$M\dot{\omega }=-D\omega +P_{m}-P_{e}$$ (2)

where *δ* is the rotor angle of the synchronous generator, *ω* is the rotor speed with respect to synchronous reference, *M* is the moment of inertia, *D* is the damping factor of the synchronous generator, *P*_*m*_ indicates the mechanical input power to the synchronous generator which is usually assumed to be constant in short-term power system stability problems, and *P*_*e*_ represents the active electrical power delivered by the synchronous generator.

According to the principle of the conservation flux of the closure windings, the rotor windings dynamic equation of the synchronous generator can be modeled by ignoring the impact of damping windings. Fig 1(B) schematically shows the fixed abc coordinate system defined by the aa', bb' and cc' axis of the stator and the dq coordinate system rotating with the rotor in a synchronous generator (d-axis centered in the rotor field's magnetic north pole N and the q-axis perpendicular to the S-N axis). Taking into account the electromagnetical interactions between the involved field, damping and stator windings given in the abc-stator coordinate system (a, b and c denoting the three stator phases), finally we have

$$E_{f}=E_{q}+T_{d0}\dot{E}$$ (3)

which is formulated in the dq-rotor coordinate by the abc → dq0 Park's transformation. Here *E* is the quadrature-axis transient voltage of the synchronous generator, *E*_*q*_ is the quadrature-axis voltage of the synchronous generator, *T*_*d0*_ represents the direct-axis open-circuit transient time constant of the synchronous generator, and *E*_*f*_ denotes the field excitation voltage.

Based on the corresponding vector relation [35], we obtain the following algebraic equations:

$$E_{q}=E+(x_{d}-x_{d}^{'})I_{d}$$ (4)

$$I_{d}=\frac{E-V_{s}\cos\delta }{x_{d\sum }^{'}}$$ (5)

$$I_{q}=\frac{V_{s}\sin\delta }{x_{q\sum }}$$ (6)

The active power *P*_*e*_ of the synchronous generator *G* is expressed as follows,

$$P_{e}=\frac{V_{s}E\sin\delta }{x_{d\sum }^{'}}$$ (7)

where *x*_*q∑*_ = *x*_*q*_*+x*_*T*_*+x*_*L*_ and *x*^*′*^_*d∑*_ *= x*^*'*^_*d*_*+x*_*T*_*+x*_*L*_, with *x*_*q*_ the quadrature-axis synchronous reactance, *x*^*'*^_*d*_ the direct-axis transient reactance, *x*_*T*_ the reactance of the transformer, and *x*_*L*_ the reactance of the transmission line. The symbols *I*_*d*_ and *I*_*q*_ indicate the direct-axis and quadrature-axis currents of the synchronous generator, respectively, and *V*_*s*_ and *V*_*t*_ represent the infinite bus voltage and the terminal voltage of the synchronous generator, respectively, as shown in Fig 1.

Substituting the electrical Eqs 4–7 into the mechanical and electrical dynamics equations of system Eqs 1–3, we obtain the complete mathematical model of the third-order SMIB power system as follows:

$$\dot{\delta }=\omega$$ (8)

$$M\dot{\omega }=-D\omega +P_{m}-\frac{V_{s}E}{x_{d\sum }^{'}}\sin\delta$$ (9)

$$T_{d0}\dot{E}=E_{f}-E+\frac{(x_{d}-x_{d}^{'})V_{s}}{x_{d\sum }^{'}}\cos\delta$$ (10)

In this paper, for convenience, we set *B* = $1/x_{d\sum }^{\prime }$ *1/ x*^*′*^_*d∑*_, *X = x*_*d*_ – − *x*_*d*_^*'*^ and renormalize the equations, yielding

$$\dot{\delta }=\omega$$ (11)

$$M\dot{\omega }=-\gamma \omega +P_{m}-BV_{s}E\sin\delta$$ (12)

$$\alpha \dot{E}=E_{f}-E+XBV_{s}\cos\delta$$ (13)

Clearly now we have three state variables *δ*, *ω* and *E* in the model, and they are expressed together as the state vector ***X*** = [*δ*, *ω*, *E*]^*T*^. The other letters including *M*, *B*, *V*_*s*_, *X*, *E*_*f*_, *α*, *P*_*m*_ and γ are all system parameters, which do not change with time. In fact, here we can factor out the inertia constant (*M*) in the left of Eq 12 completely. However, in order to investigate its effect, we keep it and make *M* as a system parameter. Recently the low-inertia stability issues including virtual inertia-emulating devices and virtual inertia placement schemes are of key importance in renewables integration to grids and power system dynamics, and thus it would be very interesting to study the effect of the inertia constant for different *M*’s [33].

Compared with the classical swing equation, which includes only Eqs 11 and 12 and treat the voltage magnitude *E* as constant (*E = 1*.*0* is chosen in the paper, without losing generality), the whole three-order model Eqs 11–13 treats *E* as a state variable, which can vary with time. In the power system field, this model more precisely characterizes the synchronous generator dynamics with excitation control.

In our simulations, we choose the mechanical input power *P*_*m*_ and the damping coefficient *γ* as our primary operation parameters, but fix all other parameters: *B = 1*.*0*, *V*_*s*_ *= 1*.*0*, *E*_*f*_ *= 1*.*0*, *α = 2*.*0* and *X = 1*.0 [25], which are typical in per-unit in power systems. The classical fixed-step fourth-order Runge-Kutta method for solving differential equations has been used with the time step being 0.01, which is small compared to the period. Therefore, below we will mainly investigate the dynamical behaviors with the variation of *P*_*m*_ and γ, and especially focus on the difference with different values and signs of the damping coefficient γ, which can be positive or negative. We are also interested in the difference with the classical second-order swing model which ignores the voltage dynamics.

## Rich Dynamics and System Collapse

### The case for positive damping

**Fig 2:**
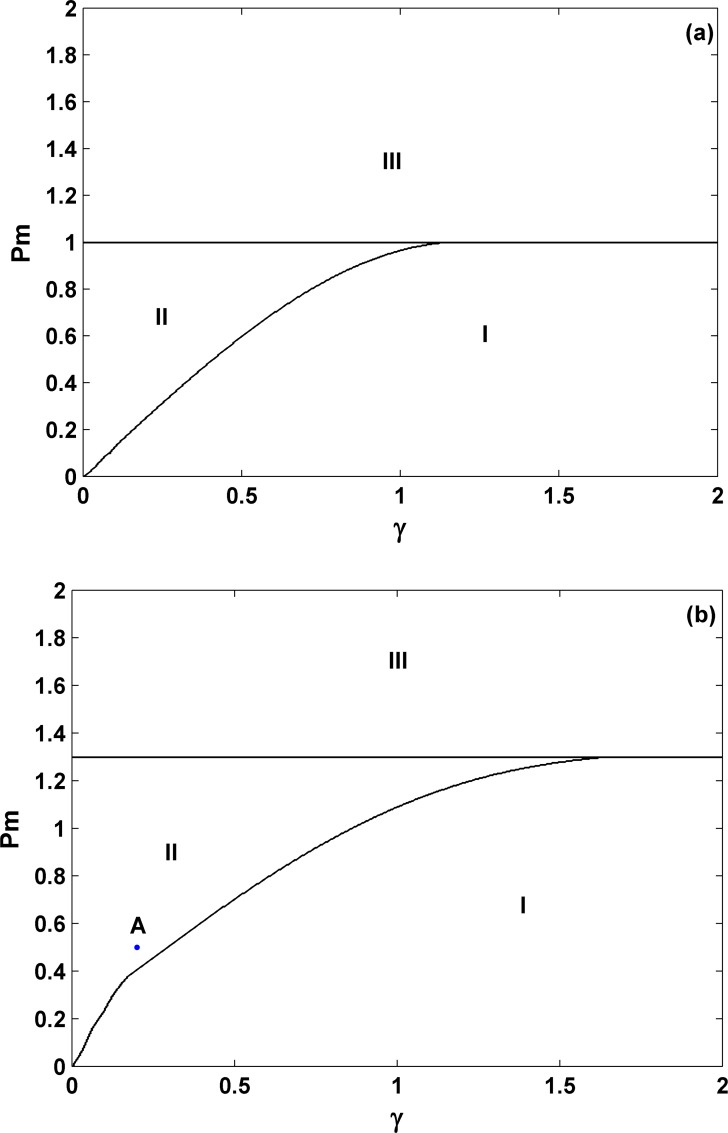
**Comparison of phase diagrams for γ>0 in the second-order power system [Eqs 11–12] (a) and the third-order power system [Eqs 11–13] (b).** Here the symbol **I** indicates that of stable fixed point, **III** denotes that of stable limit cycle, and **II** represents the area for their coexistence (namely, for different initial conditions two different attractors can appear and coexist). This comparison for qualitative similarity indicates that the voltage dynamics has not significant influence on the dynamic behaviors if the damping is positive. In 2(a), *E* = 1.0 is chosen for the second-order system throughout the whole work. In 2(b), the letter A indicates the parameters: *P*_*m*_ = 0.5 and γ = 0.2. All other parameters *M = 1*.*0*, *B = 1*.*0*, *V*_*s*_ *= 1*.*0*, *E*_*f*_ *= 1*.*0*, *α = 2*.*0* and *X = 1*.*0* are fixed. Clearly a constant critical parameter *P*_*mSNB*_ = 1.299 exists for the third-order system, compared to *P*_*mSNB*_ = 1 for the second-order one, which comes from the similar saddle-node bifurcation and can be analytically obtained.

In this section, we will mainly rely on numerical simulations by using several standard techniques in nonlinear dynamics, such as time series analysis, phase diagram, Poincare section, Lyapunov exponents, etc. First let us consider the case for the unit one inertia constant, *M* = 1, and the positive damping, γ>0. The effect of the inertia constant will be studied in the end of this section. The second-order SMIB power system [Eqs 11 and 12] with *E = 1* fixed in (12) and the third-order SMIB power system [Eqs 11–13] will be treated and compared. Fig 2(A) and 2(B) are the phase diagrams for different dynamic behaviors in the *P*_*m*_- γ parameter space for these two systems, respectively. Simply we use **I** to indicate the area of stable fixed point, **III** for stable limit cycle, and **II** for their coexistence. Based on these observations, although the precise values for different types of dynamics are different, their qualitative behaviors are similar [see the three major areas and compare the different locations of the critical curves in the figures]. In the whole parameter region, no more complicated behavior, such as quasi-periodic, can be found. Therefore, we may conclude that if the damping is positive, considering the voltage dynamics may have not significant influence on the corresponding dynamics. Note that these three different types of dynamics in the second-order system have been well recognized and studied in different fields, such as in power system dynamics, in mechanics for forced pendulum, and in physics for Josephson junctions, etc [13].

**Fig 3:**
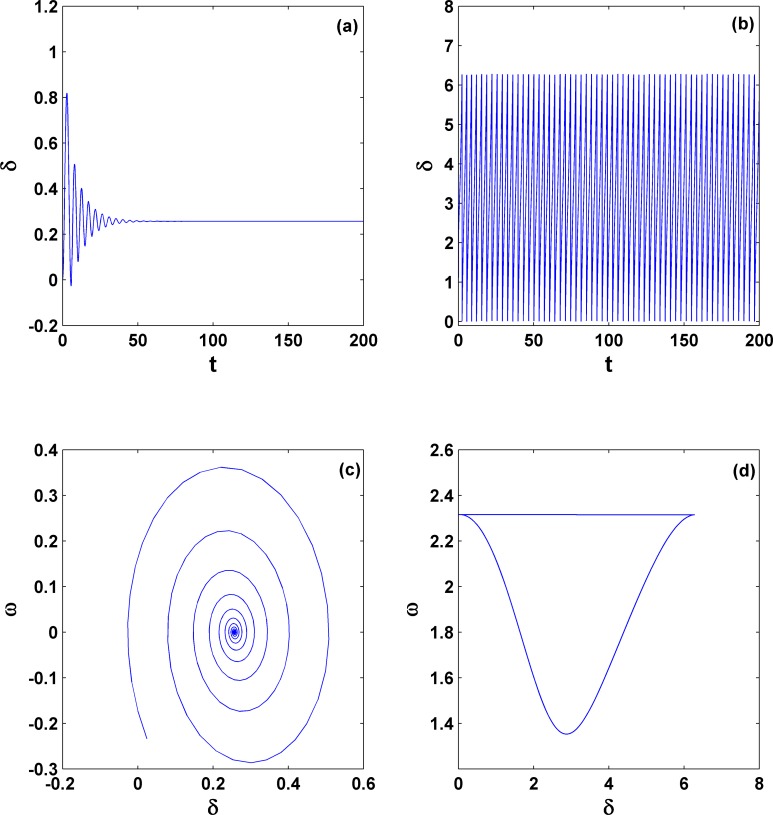
Coexistence dynamics for different initial conditions. They are stable fixed point [(a) and (c)] and stable limit cycle [(b) and (d)] with the parameters (*P*_*m*_ = 0.5 and γ = 0.2) [denoted by the letter A within area **II** in Fig 2(B) for the third-order system]. They are simulated with the same parameters but two different initial conditions. This coexistence can also be extensively found in the second-order system under the condition of positive damping. In (d), the angle is treated as mod 2π.

To show the similarity to the second-order system in detail, we choose the parameter set (*P*_*m*_ = 0.5 and γ = 0.2) [indicated by point A within the coexistence region in Fig 2(B)] and study its dynamics. The results are shown in Fig 3, where the left panels [(a) and (c)] are for the stable fixed point and the right panels [(b) and (d)] are for the stable limit cycle, depending only on their initial conditions.

**Fig 4:**
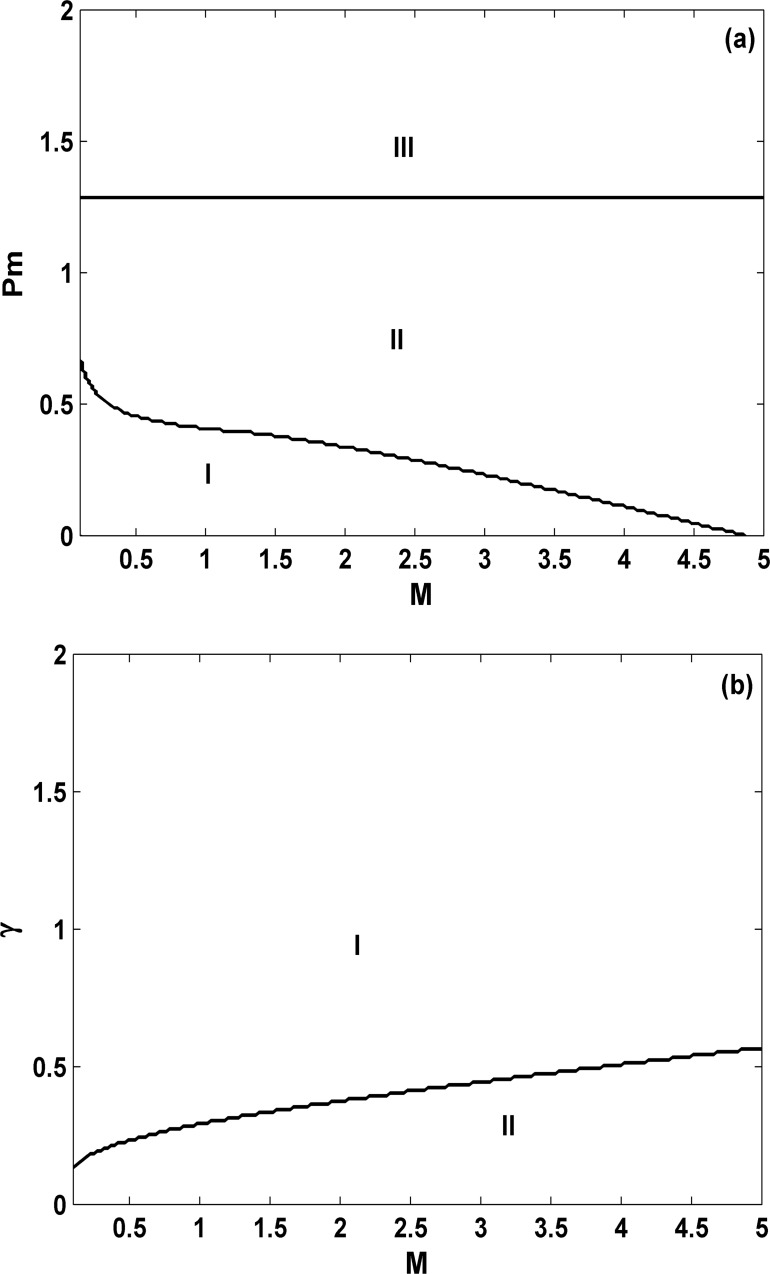
**Phase diagrams on the *M*-*P***_***m***_ **parameter plane; γ = 0.2 (a) and on the *M*- γ parameter plane; *P***_***m***_ **= 0.5 (b) in the third-order power system.** Again similar dynamics including stable fixed point (I), stable limit cycle (III) and their coexistence (II) are found with the change of *M*. Hence with the increase of *M*, the coexistence region increases gradually and the dominant stable fixed point region decreases correspondingly. This indicates that smaller *M* favors the occurrence of stable fixed point.

Next let us consider the inertia effects in the third-order power system for different *M*’s in Fig 4. The parameter γ = 0.2 is fixed in Fig 4(A) and *P*_*m*_ = 0.5 is fixed in Fig 4(B). Clearly three different types of behavior including stable fixed point (**I**), stable limit cycle (**III**) and their coexistence (**II**) persist with the change of *M*. In addition, we find that the region of **I** for the dominant stable fixed point gets larger with smaller *M* in both Fig 4(A) and 4(B) for either fixed γ or *P*_*m*_. Further simulations show that the transient behavior for different inertia constants can be entirely different. For example, for *M = 0*.*03*, both *δ* and *E* very fast damp to the equilibria due to smaller inertia, whereas for *M = 4*.*0*, their transient trajectories become oscillatory and slow; *P*_*m*_ = 0.5 and γ *= 0*.*2* are fixed. Therefore, we know that although the inertia constant does not affect the equilibria, which can be verified in the theoretic analysis part, but their local stability properties and the transient behavior are entirely different. On the other hand, it is well-known that robustness to disturbance is more important than a large parameter region of attraction, in term of proper operation of power systems. Thus further parameter sensitive analysis and even dynamical analysis under either noise or intentional disturbance are imperative for our deeper understanding of inertia effects on such a classical third-order flux decay equation.

### The case for negative damping

Now we will study dynamic behaviors for negative damping (*M* = 1 fixed first without losing generality). As we have stated in the introduction, many researchers have considered the positive damping effect and investigated the related excitation control. Several decades ago, however, Concordia and Carter have analyzed the negative damping of electrical machinery and proposed that the negative damping coefficient is related to the generator angle and the armature resistance [36]. Based on the fact that these features determine the property of the generator, the negative damping will play a significant role in the stability of the synchronous generator and further of the whole power systems. Thus it becomes very crucial to study the negative damping effect in power system dynamics. Below we will see that with the consideration of voltage dynamics, the system dynamics indeed becomes much more complicated and distinctive, compared with the classical second-order model.

**Fig 5:**
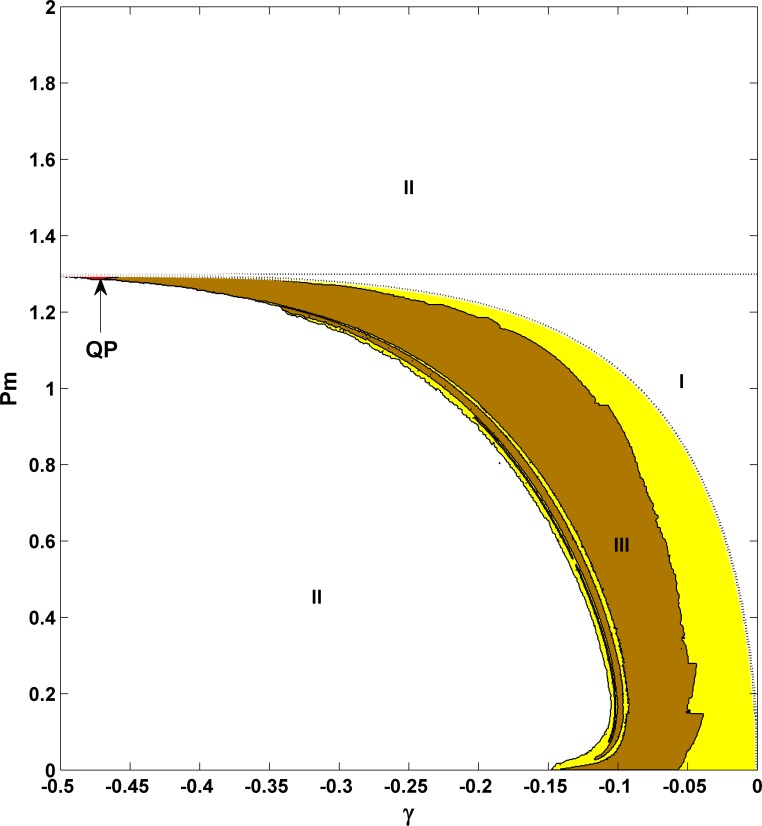
Phase diagram for γ<0 in the third-order power system. Here area **I** indicates stable fixed point, **II** represents explosive solutions for system collapse, and **III** shows very rich dynamics, such as periodic orbits (orange-yellow), chaos (yellow), and quasi-periodic orbits (red and at the left corner of **III**, indicated by the capital letters QP). In addition, the theoretic results for the saddle-node bifurcation (the horizontal line) and the supercritical Hopf bifurcation (the curved line) have been superimposed with two dashed lines, showing perfect fits with the numerical results. Clearly a constant critical parameter *P*_*mSNB*_ = 1.299 still exists, the same as in Fig 2(B) for γ>0. In a sharp contrast to all these, the second-order power system is quite simple; the whole parameter space is occupied by the explosive solution.

Fig 5 illustrates the phase diagram in the *P*_*m*_- γ parameter space for the third-order system with γ<0 (*M* = 1), where it can be roughly divided into three areas again. Now we use **I** to indicate stable fixed point, **II** to represent system collapse, and **III** to show very complicated dynamical behaviors with different colors including periodic orbits (orange-yellow), quasi-periodic orbits (red), and chaos (yellow). The area of quasi-periodic motions is located only in the small region of the left corner of area **III**. As there are periodic windows in the chaotic regions, the areas for periodic orbits and chaotic orbits are scattered and interlaced within area **III**. We will go to more details in the subsequent parts.

**Fig 6:**
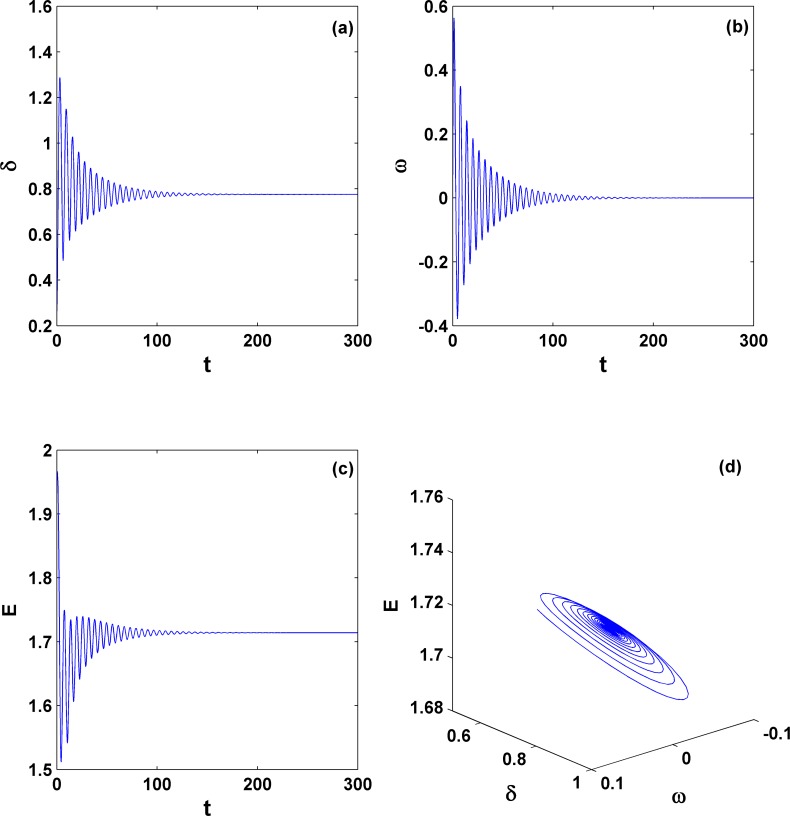
Dynamical behavior of the stable equilibrium state. The time-series diagrams [(a)-(c)] and trajectory diagram in the phase space (d) for parameters within area **I** in Fig 5(B); *P*_*m*_ = 1.2 and γ = −0.117. Clearly here the equilibrium state persists for negative damping; this is one of key differences with the second-order system.

For the sake of brevity, several different parameter values corresponding to the typical dynamic behaviors are selected within different parameter areas. For example, within area **I** as the mechanical power *P*_*m*_ and the damping coefficient γ equal 1.2 and −0.117, respectively, the system clearly shows a stable fixed point (Fig 6). This finding verifies that we can still find a large parameter region outside of the traditional region for γ>0 as shown in Fig 2. This new region for a stable equilibrium point with negative damping is of great significance for our understanding of dynamical behaviors and proper operation in power systems, and it can be viewed as one key difference to the second-order power system.

**Fig 7:**
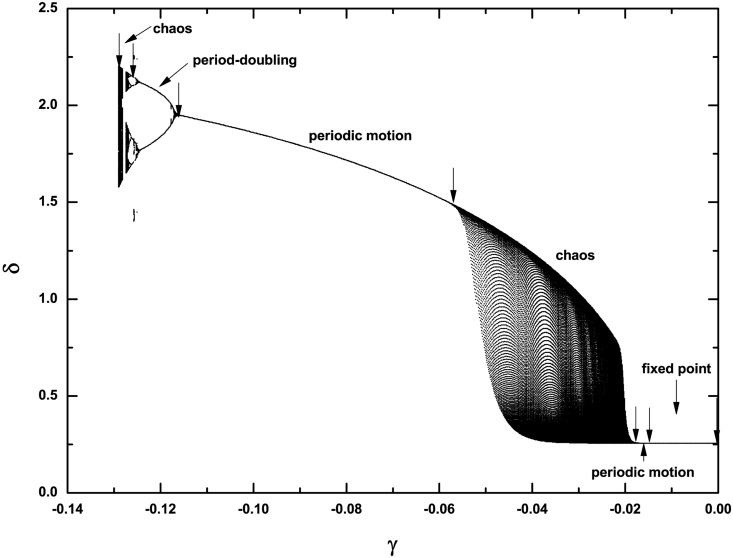
Bifurcation diagram for the damping coefficient γ. *P*_*m*_ = 0.5. In the figure, from right to left: the transition scenario is from fixed point → periodic motion → chaos → periodic motion → period-doubling cascade → chaos → collapse. The parameter regions are indicated by the arrows here.

**Fig 8:**
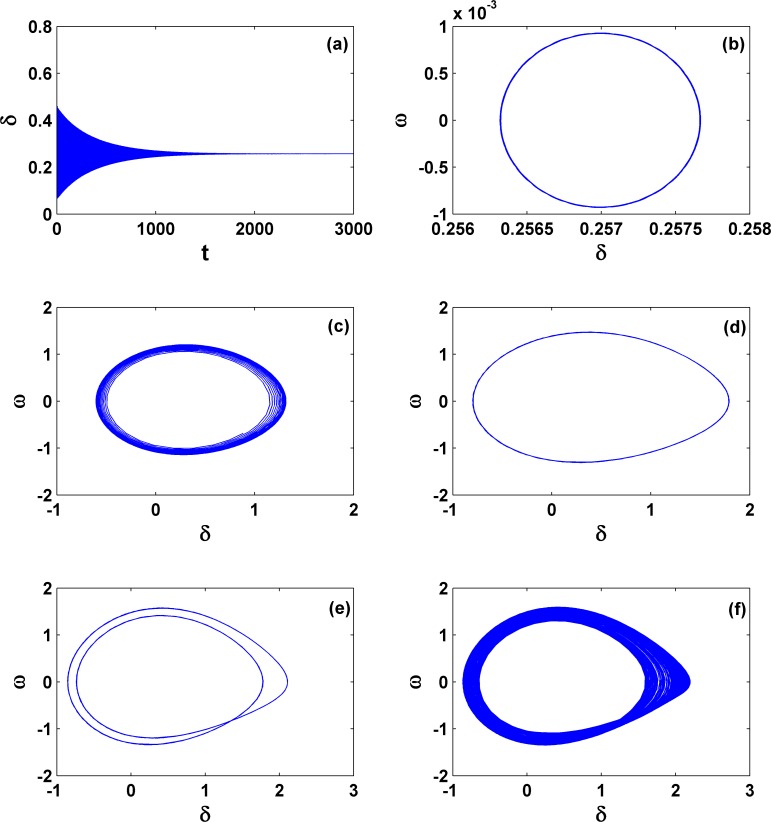
Different dynamical behaviors with the change of γ, corresponding to Fig 7. From (a) to (f), the parameter γ^′^s are −0.01, −0.0152, −0.045, −0.09, −0.124 and −0.129, respectively, and *P*_*m*_ = 0.5 is unchanged. In (c) and (f), the system shows chaotic motion, and from (d) to (f), the system exhibits a period-doubling route to chaos.

Next let us concentrate on the dynamics within area **III** and see how the stable fixed points within area **I** become unstable. Without losing generality, we fix *P*_*m*_ = 0.5 and change the values of γ. The bifurcation diagram with the extreme value of the time series *δ(t)* for changing γ is shown in Fig 7, which exhibits two pieces of periodic and chaotic regions but with clearly different nature. From right to left, with a decrease of γ, the first region of periodicity happens only within a very tiny parameter region, as indicated by the arrow, after the immediate instability of the fixed point. The first region of chaos happens from the instability of fixed points and subsequently from the instability of periodic orbits. Our analysis shows that the fixed point within area **I** becomes unstable via a supercritical Hopf bifurcation and this critical loci from area **I** to area **III** can be analyzed, as we will show later. In contrast, the second region for chaos happens due to a period-doubling cascade. Therefore, the whole route becomes: fixed point → periodic motion → chaos → periodic motion → period-doubling cascade → chaos. These typical types of behavior are shown in Fig 8 for several different γ's; from 8(a) to 8(f), γ = −0.01, −0.0152, −0.045, −0.09, −0.124 and −0.129. In Fig 8(B), the size of the periodic motion is very tiny, whereas that in Fig 8(D) begins becoming normal. In addition, these periodic motions correspond to an oscillation in the neighborhood of an equilibrium point, which is fundamentally different with the observation of a periodic orbit in Fig 3(D), where the angle is treated as mod 2π, corresponding to a periodic rotation of the pendulum. Obviously chaos appears in 8(c) and 8(f). This means that in the three-order power system by including voltage dynamics, chaos becomes very typical if the negative damping coefficient has been properly chosen. This can also be viewed as one key difference with the second-order power system.

**Fig 9:**
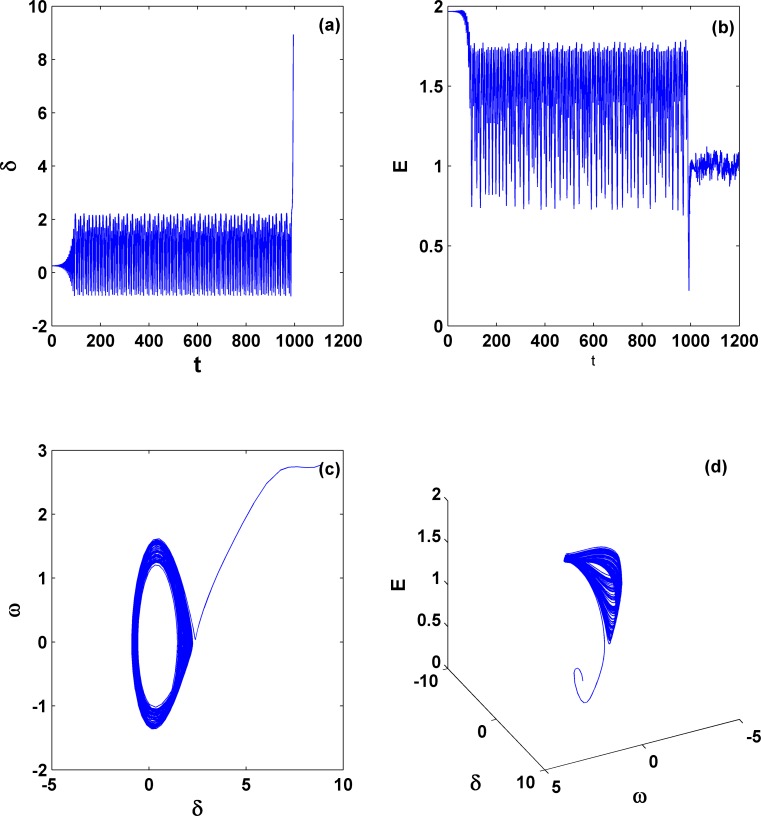
System collapse due to boundary crisis. The parameters *P*_*m*_ = 0.5 and γ = −0.1304 are chosen quite close to but outside of the stable chaotic region (i.e., the region near the critic curve for dividing areas **II** and **III** in Fig 5). Clearly here the system exhibits chaos in the transient process, and *E* still keeps a finite value after the transient.

With further decreasing γ, the chaotic motion may also become unstable and the system collapses. As an example, Fig 9 shows such an instability for γ = −0.1304. It is clear that the system collapses after a very long transient time with a chaotic behavior. This pattern for the disappearance of a chaotic attractor and the accompanying system collapse usually appears when a strange chaotic attractor collides with another unstable limit set, which is probably an unstable limit cycle or a saddle. In the literature, it is generally called boundary crisis [37, 38]. In addition, we find that the system collapses for the phase variables (*δ* and *ω*) only, whereas the voltage magnitude (*E*) still keeps a nearly constant value after the transient. Later we will see this type of partial collapse is very common in this model.

**Fig 10:**
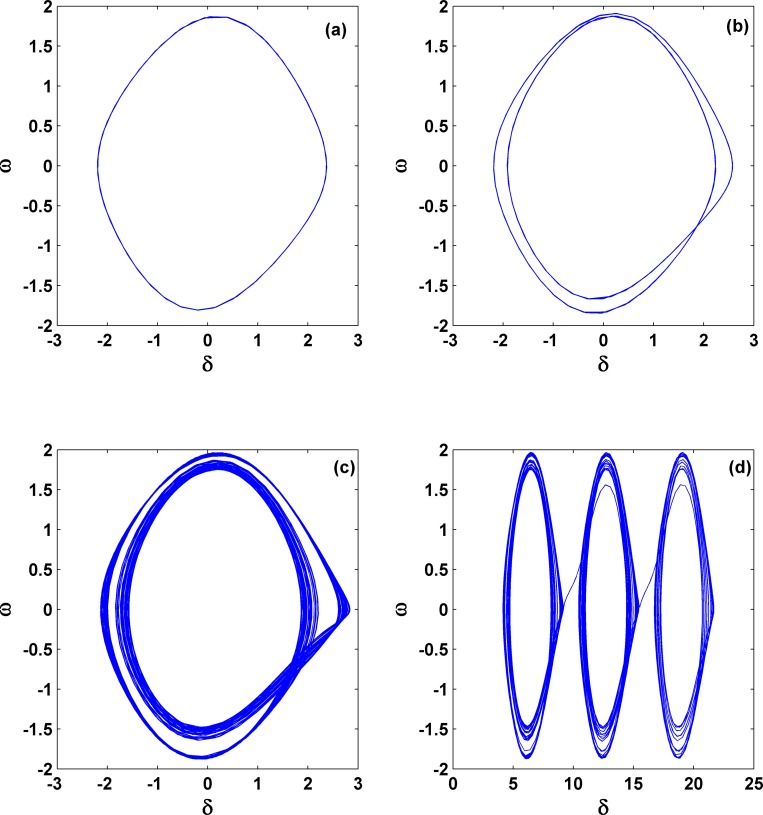
Different dynamical behaviors with the change of Pm. From (a) to (d), the parameter *P*_*m*_'s are 0.01, 0.024, 0.035, and 0.0375, respectively; *γ* = −0.1 γ = −0.117 is unchanged. From (a) to (c), the route from period-doubling bifurcation to chaos is clear. In (d), an unusual periodic chaos appears, showing chaotic motion in each rotation but nearly periodic motion between these big rotations. This indicates that different with the boundary crisis shown in Fig 9, an un-collapse periodic chaos behavior, similar to chaotic scroll attractors with infinite scrolls, may still appear.

Next in order to exhibit the generality of the route to chaos by period-doubling bifurcation within the area **III**, we study the dynamics with the increase of the mechanical power *P*_*m*_ but let the damping coefficient unchanged (γ = −0.117). The results are shown in Fig 10, with period-1, period-2, and chaotic motions presented in Fig 10(A), 10(B) and 10(C), respectively. Correspondingly, *P*_*m*_ = 0.01, 0.024, and 0.035. These panels prove that the period-doubling scenario transition to chaos is very common in the third-order system for negative damping. In addition, to our surprise, with the increase of *P*_*m*_ and still within the area **III**, we find another unusual behavior; it is still chaotic in each piece of attractor but nearly periodic between these pieces. This phenomenon is exemplified in Fig 10(D) for *P*_*m*_ = 0.0375. We may call it *periodic chaos*. Detailed calculation for the equilibrium points and their stabilities shows that the first piece of chaotic attractor rotates around one equilibrium point, which is unstable, and after several rotations it suddenly collides with the other equilibrium point, which is also unstable, and jumps to the next piece of the chaotic attractor. This phenomenon can extend further with time. Thus this type of behavior is similar to the multi-scroll attractors [39, 40], such as the well-known Chua’s double scroll, where multiple equilibrium points in the scroll-based chaotic attractor by modifications of nonlinear characteristics are necessary.

**Fig 11:**
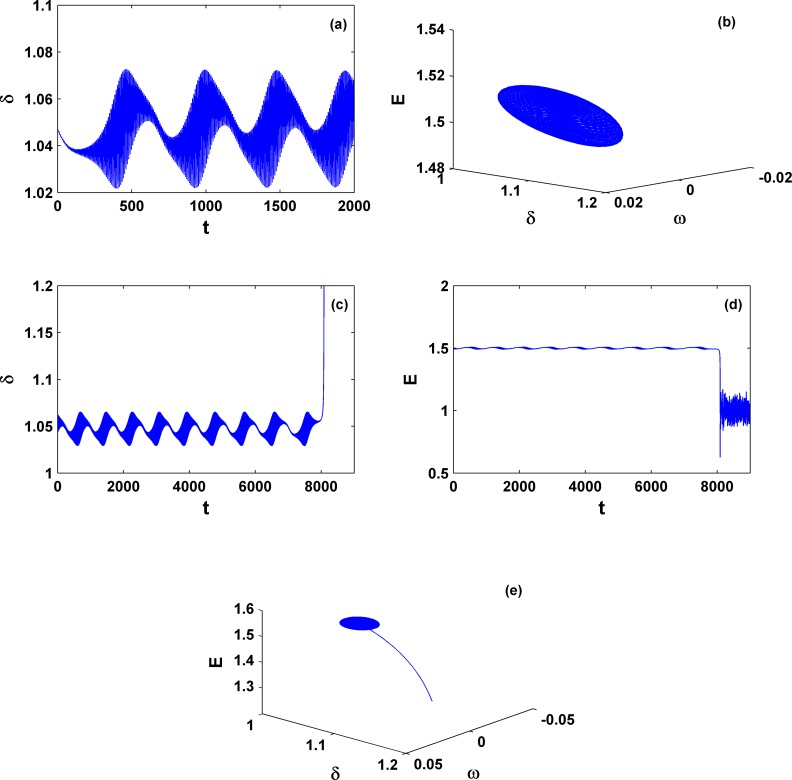
Variation of dynamical behaviors. (a) and (b) Dynamics for stable quasi-periodic motion (*P*_*m*_ = 1.2989 and γ = −0.49991), whereas (c), (d), and (e) for unstable quasi-periodic motion (*P*_*m*_ = 1.298968 and γ = −0.50001). These two parameter sets are very close and both are located around the left corner of area **III**. This comparison indicates that different with the system collapse form chaos due to boundary crisis, as shown in Fig 9, the other form of collapse from quasi-periodic motion still exists.

Relying on the calculation of the Lyapunov exponents and dynamical analysis, still within the area **III** we can even find another type of dynamical behavior, quasi-periodic motion, which appears only in a very tiny parameter region close to *P*_*m*_ = 1.3 and γ = −0.5. To be clearer, we use the symbols QP in Fig 5 to denote it. The corresponding dynamics is shown in Fig 11(A) and 11(B); *P*_*m*_ = 1.2989 and γ = −0.49991.

In addition, we find that this quasi-periodic motion can become unstable. See, e.g., the pattern in Fig 11(C), 11(D) and 11(E) for *P*_*m*_ = 1.298968 and γ = −0.50001. Clearly this is another type of collapse, which is different from the usual collapse of chaos. However, we find again that the phase variables (*δ* and *ω*) go infinity immediately and the voltage magnitude (*E*) remains finite after the transient. This observation may not be a surprise; actually it can be proven by the applications of variations of constant formula in Eq 13.

**Fig 12:**
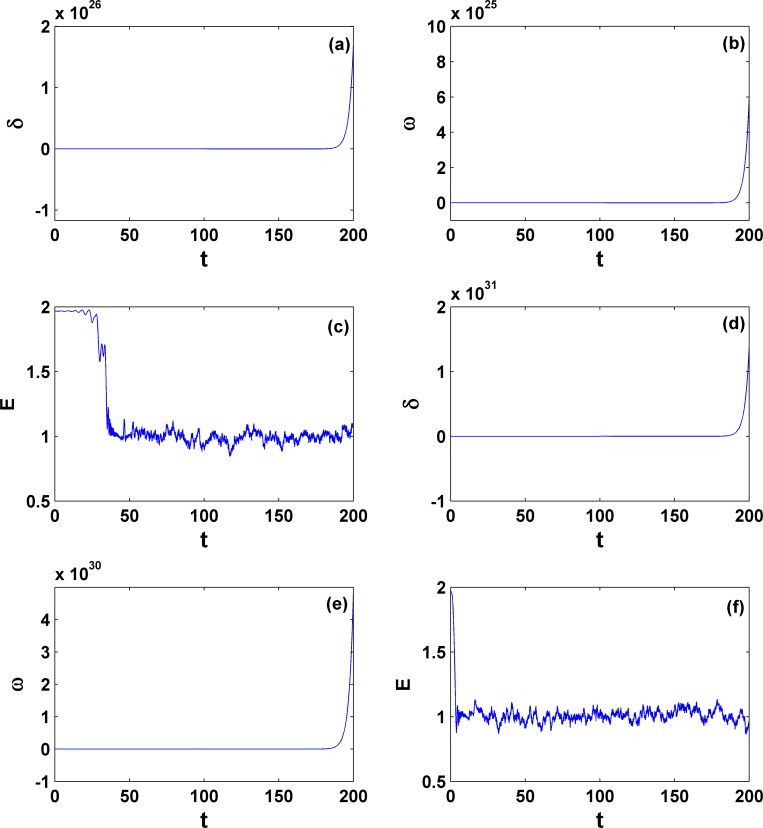
Dynamics for system collapse within area II. The parameters are *P*_*m*_ = 0.5 [(a), (b), and (c)] and *P*_*m*_ = 1.4 [(d), (e), and (f)]; γ = −0.35. Similarly the rotor-angle instability is dominant and the voltage remains stable with its magnitude keeping within a finite value region.

Furthermore, let us move on the general behavior within the area **II**. Without losing generality, two different parameter sets are chosen, one within the lower **II** and the other within the upper **II** and both are far from the critical curves. The explosive behaviors for a partial system collapse for the phase variables only are expected there. Indeed we find that now the phase (rotor-angle) *δ* goes to infinity after a very short transient, whereas the variable *E* keeps finite but fluctuates, as shown in Fig 12. This phenomenon demonstrates that although the third-order model incorporating voltage variation is studied here, it can only exhibit rotor-angle instability similar to what the classical second-order model can show. For the occurrence of a real collapse of voltage magnitude, the load dynamics has to be further added and considered.

**Fig 13:**
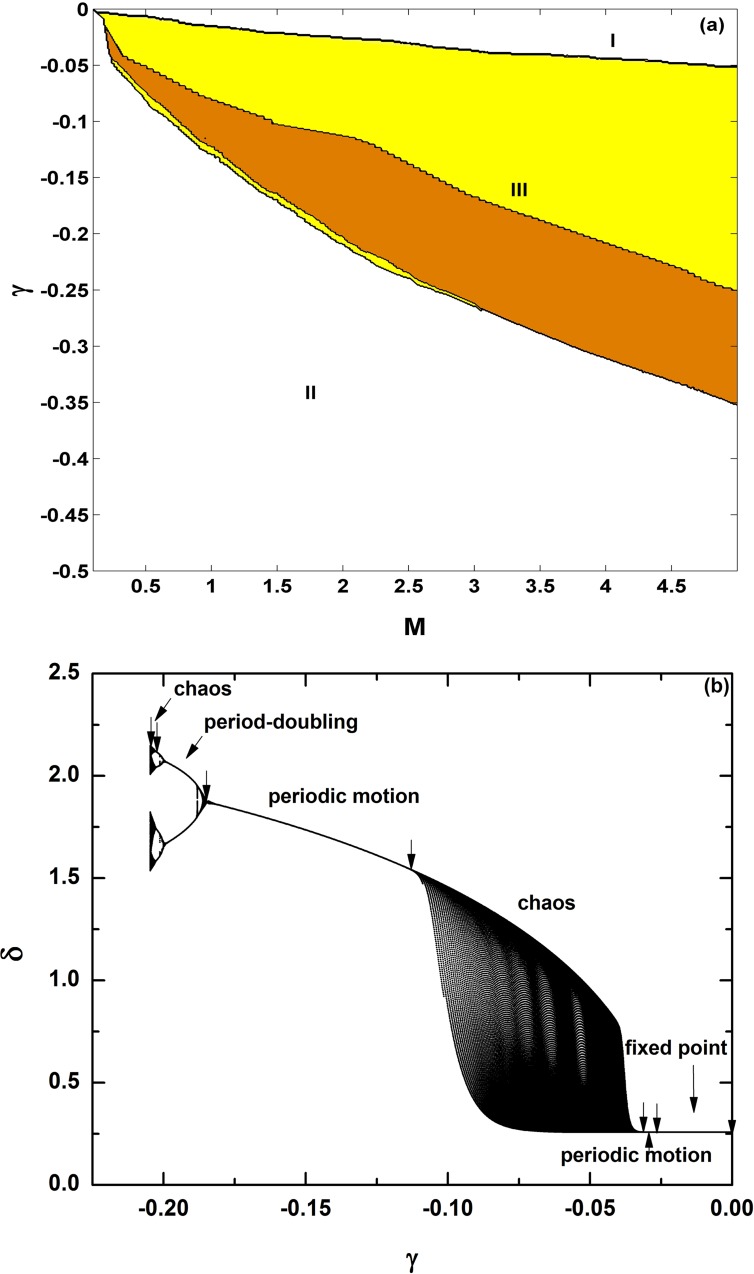
**Phase diagram on the *M*- γ parameter plane; *P***_***m***_ **= 0.5 and γ<0 (a) and bifurcation diagram with the change of damping coefficient γ; *M* = 2.0 (b) in the third-order power system.** Again in (a), area **I** indicates stable fixed point, **II** represents explosive solutions for system collapse, and **III** shows very rich dynamics, such as periodic orbits (orange-yellow), chaos (yellow). In (b), from right to left: the transition scenario is similar, from fixed point → periodic motion → chaos → periodic motion → period-doubling cascade → chaos → collapse at γ = −0.2087. We can find that opposite to the case for positive damping in Fig 4, now larger inertia constant *M* enlarges the stable fixed point area **I**.

Finally, similar to the study in Fig 4 for positive damping, let us consider and study the inertia effects in the third-order power system for negative damping. The phase diagram on the *M-* γ parameter space is shown in Fig 13(A); *P*_*m*_ = 0.5. As one example, the bifurcation diagram for a fixed *M* (*M* = 2.0) is shown in Fig 13(B). Again area **I** indicates a stable fixed point, **II** represents explosive solutions for system collapse, and **III** shows rich dynamics, such as periodic orbits (orange-yellow), and chaos (yellow). From right to left in Fig 13(B), with decreasing *γ*, the system appears a variety of dynamic behavior, from fixed point → periodic motion → chaos → periodic motion → period-doubling cascade → chaos → collapse, except that now the system collapses at γ = −0.2087. Comparing these diagrams with those in Figs 5 and 7 for *M* = 1, we find that the behavior is quite similar. Observing Fig 13(A), however, we find that the region for area **I** for the stable fixed point becomes larger with the increase of *M*; this behavior is quite different to that for positive damping in Fig 5, where smaller inertia favors the stable fixed point, in term of the size of stable parameter region of fix points. This difference can be regarded as one key distinction induced by inertia.

**Fig 14:**
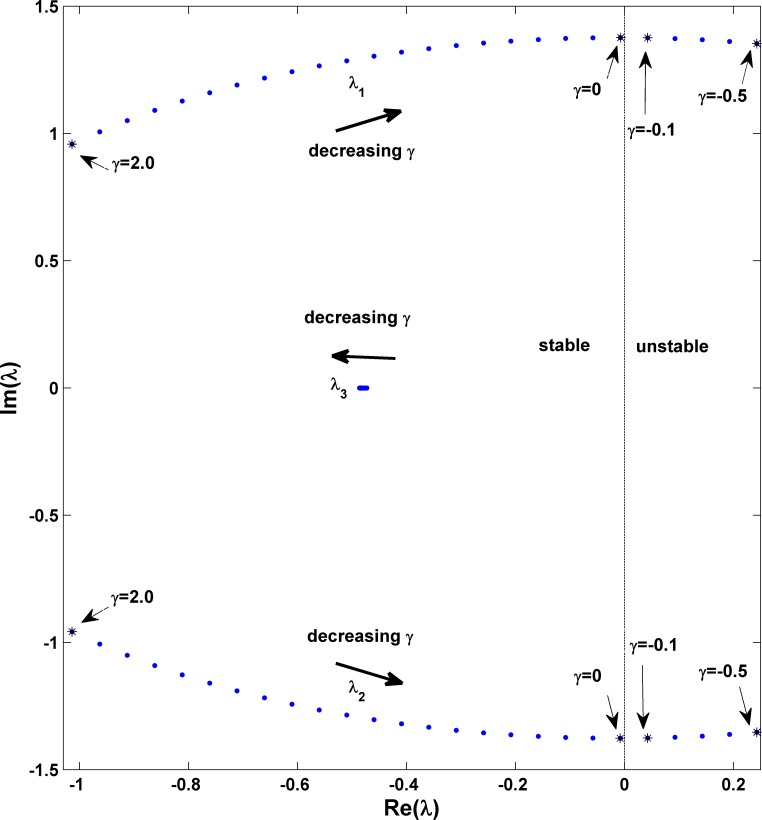
Modal analysis showing the loci of eigenvalues of fixed point with the change of γ; *P*_*m*_ = 0.5 *and M* = 1.0 are fixed. We can find that with decrease of γ from γ = 2.0 to γ = −0.5 with Δγ = −0.1, a pair of leading characteristic values (λ_1_ and λ_2_) comes across the imaginary axis from the left (stable region) to right (unstable region) at γ = −0.0151, indicating that the fixed point becomes unstable due to a super-critical Hopf bifurcation. This is coincident with our observation in Fig 5 for the occurrence of stable fixed point region even when the negative damping is considered. However, more complicated behavior within area **III** can only be available with nonlinear analysis.

In the power system dynamics field, engineers are quite familiar with the technology of modal analysis in studying small-signal stability (or called small-disturbance stability) [2]. Therefore, it would be very interesting to view the behavior of the eigenvalues of the fixed point as the damping moves from positive to negative. The result of this change for different system parameters γ from γ = 2.0 to γ = −0.5 with Δγ = −0.1 in the eigenvalue space is shown in Fig 14, where it is clear that a pair of leading eigenvalues (λ_1_ and λ_2_) comes across the imaginary axis from left to right at the bifurcation point γ = −0.0151, whereas the other eigenvalue (λ_3_) remains real and negative. This value of bifurcation point coincides with that in the phase diagram in Fig 5, and this type of behavior is indicative of a super-critical Hopf bifurcation. In the theoretic analysis part, we will further obtain more details in a more mathematical manner. In addition, we know that the fixed point is always locally stable under the condition of positive damping (γ>0); this point is also in accordance with the observation in Fig 2(B). It is apparent that with this local linearization method of the fixed point, we can only obtain local stability or instability information but we cannot get further information for parameters within the region of complex behavior in Fig 5.

## Theoretic Analysis

So far, based on numerical simulations we have obtained exhausted observations and have found several unusual behaviors, such as the stable fixed point, chaos, periodic motion, quasi-periodic motion, and their collapses when a negative damping parameter is considered. Apparently their appearance fundamentally contradicts with our common sense that negative damping can only disrupt the system, such as making the rotor-angle diverge in either oscillating or monotonic manner in power systems. In particular, the newly born region of equilibrium state is also of special interest from the power electric engineering point of view, as it is a novel type of phenomenon when the third-order model incorporating voltage dynamics is considered. Hence it becomes valuable to further understand such an unusual behavior mathematically, which will be the main objective of this section. For convenience of theoretical analysis, we take *M = 1*.*0*.

### The saddle-node bifurcation

According to Eqs 11–13, the equilibrium points for the system are the solutions of the following set of algebraic equations:

0=ω (14)

$$0=-\gamma \omega +P_{m}-BV_{s}E\sin\delta$$ (15)

$$0=E_{f}-E+XBV_{s}\cos\delta$$ (16)

Based on the first two equations [Eqs 14 and 15], we immediately know that all the equilibrium points are determined by the mechanical power *P*_*m*_ only and they are unrelated to the damping coefficient *γ*.

Further we can obtain how the two variables *E* and *δ* and *δ* are related to the only adjustable parameter *P*_*m*_, respectively, as follows:

$$P_{m}^{2}=\frac{E^{2}}{X^{2}}(B^{2}V_{s}^{2}X^{2}-E^{2}-E_{f}^{2}+2EE_{f})$$ (17)

$$P_{m}=BV_{s}E_{f}\sin\delta +XB^{2}V_{s}^{2}\cos\delta \sin\delta$$ (18)

The functional forms in the above two equations are very complicated, and therefore, we cannot have the explicit expressions for *E* and *δ* as a function of *P*_*m*_. Basically they have two positive solutions for *E* and *δ*, respectively, and the system will have two equilibrium points. Their relation can be stated as follows:

1. If 0 ≤ *P*_*m*_ < *P*_*mSNB*_, then the system has exactly two equilibrium points denoted as ***X***_*s*_ *= (δ*_*s*_,*0*,*E*_*s*_*)* and ***X***_*u*_ *= (δ*_*u*_,*0*,*E*_*u*_*)*, where ***X***_*s*_ is the stable equilibrium point, and ***X***_*u*_ is the unstable one with *δ*_*s*_<*δ*_*u*_ and *E*_*s*_>*E*_*u*_;
2. If *P*_*m*_ = *P*_*mSNB*_, then the system has only one equilibrium point with ***X***_*SNB*_ *= (δ*_*SNB*_,*0*,*E*_*SNB*_*)*. The parameter point *P*_*m*_ = *P*_*mSNB*_ is the so-called static bifurcation point in power system dynamics;
3. If *P*_*m*_>*P*_*mSNB*_, then these two equilibrium points collide and annihilate, indicative of a saddle-node bifurcation. After several algebraic manipulations, we specifically get

$$P_{mSNB}=\frac{(3E_{f}+\sqrt{E_{f}^{2}+8B^{2}X^{2}V_{s}^{2}})\sqrt{4B^{2}X^{2}V_{s}^{2}-E_{f}^{2}+E_{f}\sqrt{E_{f}^{2}+8B^{2}X^{2}V_{s}^{2}}}}{8\sqrt{2}X}$$ (19)

For the parameters used in the paper: *B = 1*.*0*, *V*_*s*_ *= 1*.*0*, *E*_*f*_ *= 1*.*0*, *α = 2*.*0*, and *X = 1*.*0*, we have the predicted value for *P*_*mSNB*_: *P*_*mSNB*_ = 1.299. *P*_*mSNB*_ is independent of γ, and this value exactly fits with our observations [see the horizontal lines for both γ>0 and γ<0 in the phase diagrams in Figs 2(B) and 5, respectively].

Correspondingly, we have the explicit expressions for several special values of the rotor angle *δ* and the transient voltage *E* for the saddle-node bifurcation, such as *δ*_*min*_, *δ*_*max*_, *δ*_*SNB*_, *E*_*min*_, *E*_*max*_, and *E*_*SNB*_, where *δ*_*min*_ and *δ*_*max*_ (*E*_*min*_ and *E*_*max*_) are minimal and maximal values of *δ* (*E*), coming from the solution in Eqs 17 and 18. With the stable branch *δ*_*s*_*∈(δ*_*min*_,*δ*_*max*_*)*, *E*_*s*_*∈*(*E*_*SNB*_, *E*_*max*_) and the unstable branch *δ*_*u*_*∈(δ*_*min*_,*δ*_*max*_*)*, *E*_*u*_*∈(E*_*min*_, *E*_*SNB*_*)*, we obtain

$$\delta_{\min}=0$$ (20)

$$\delta_{\max}=\pi$$ (21)

$$\delta_{SNB}=\arccos(\frac{-E_{f}+\sqrt{E_{f}^{2}+8B^{2}X^{2}V_{s}^{2}}}{4XBV_{s}})$$ (22)

$$E_{\min}=E_{f}-XBV_{s}$$ (23)

$$E_{\max}=E_{f}+XBV_{s}$$ (24)

$$E_{SNB}=\frac{3E_{f}+\sqrt{E_{f}^{2}+8B^{2}X^{2}V_{s}^{2}}}{4}$$ (25)

and know the solution regions for the stable or unstable equilibrium points.

### The super-critical Hopf bifurcation

The system Jacobin matrix based on the linearized equation can be stated below:

$$J=\left(\begin{array}{ccc} 0 & 1 & 0\\ -BV_{s}E_{0}\cos\delta_{0} & -\gamma & -BV_{s}\sin\delta_{0}\\ -\frac{XBV_{s}}{\alpha }\sin\delta_{0} & 0 & -\frac{1}{\alpha } \end{array}\right)$$ (26)

where the values of *δ*_*0*_ and *E*_*0*_ for equilibrium points are determined by the algebraic equations [Eqs 14–16].

Solving the characteristic equation for the above Jacobin matrix, we yield

$$\lambda^{3}+(\gamma +\frac{1}{\alpha })\lambda^{2}+(\frac{\gamma }{\alpha }+BV_{s}E_{0}\cos\delta_{0})\lambda +\frac{BV_{s}}{\alpha }(E_{0}\cos\delta_{0}-XBV_{s}\sin^{2}\delta_{0})=0$$ (27)

for the characteristic value (eigenvalue) ***λ***.

Based on the observations of the instability of the equilibrium points and the immediate appearance of limit cycles within the negative damping region in Fig 5 (see the division curve between areas **I** and **III**), we may check the condition for such a possible Hopf bifurcation, where usually in the eigenvalue space a pair of characteristic values comes across the imaginary axis from left to right with other characteristic values remaining negative in their real parts.

Assuming the characteristic equation above has the following form:

$$(\lambda +a)(\lambda^{2}+b^{2})=0$$ (28)

we obtain

$$\begin{array}{l} \lambda^{3}+(\gamma +\frac{1}{\alpha })\lambda^{2}+(\frac{\gamma }{\alpha }+BV_{s}E_{0}\cos\delta_{0})\lambda +\frac{BV_{s}}{\alpha }(E_{0}\cos\delta_{0}-XBV_{s}\sin^{2}\delta_{0})\\ =\lambda^{3}+a\lambda^{2}+b^{2}\lambda +ab^{2} \end{array}$$ (29)

which produces the following equities:

$$a=(\gamma +\frac{1}{\alpha })$$ (30)

$$b^{2}=(\frac{\gamma }{\alpha }+BV_{s}E_{0}\cos\delta_{0})$$ (31)

$$ab^{2}=\frac{BV_{s}}{\alpha }(E_{0}\cos\delta_{0}-XBV_{s}\sin^{2}\delta_{0})$$ (32)

Further we have

$$\left(\gamma +\frac{1}{\alpha })(\frac{\gamma }{\alpha }+BV_{s}E_{0}\cos\delta_{0})=\frac{BV_{s}}{\alpha }(E_{0}\cos\delta_{0}-XBV_{s}\sin^{2}\delta_{0}\right)$$ (33)

which can be simplified to

$$\gamma^{2}+(\alpha BV_{s}E_{0}\cos\delta_{0}+\frac{1}{\alpha })\gamma +XB^{2}V_{s}^{2}sin^{2}\delta_{0}=0$$ (34)

This is the exact condition for a Hopf bifurcation.

Obviously these theoretic results help us to understand the dynamical behaviors after the static bifurcation. To prove them quantitatively, we plot these predicted values as the critical curves with two dashed lines in Fig 5: one for the saddle-node bifurcation with the horizontal line and the other for the supercritical Hopf bifurcation with the curved line; their fit with the numerical results in the original phase diagram is perfect. Further calculation can prove that the Hopf bifurcation belongs to the supercritical type. The Hopf bifurcation showing the physical picture for a pair of characteristic eigenvalues comes across the imaginary axis from left to right accompanying with the critical parameter, γ = −0.0151 in Fig 14, can also be well confirmed by this analysis. In addition, all these bifurcation analysis results including the saddle-node bifurcation and the supercritical Hopf bifurcation have been confirmed by the ordinary differential equation bifurcation analysis software MATCONT in MATLAB [41].

## Conclusions and Discussions

In conclusion, nonlinear dynamic properties of the third-order SMIB power system with the classical flux decay generator model have been investigated in detail and in particular damping effects with both positive and negative damping coefficients have been considered. We have mainly found on the one hand, when the damping is positive, the third-order system and the second-order one are quite similar, exhibiting only three distinct types of dynamical behavior: stable fixed point, stable limit cycle, and their bistability. Therefore, even in this third-order differential equation, positive damping cannot induce quasi-periodicity or chaos. On the other hand for the parameter region for negative damping, we have uncovered very rich and complicated dynamics, such as stable fixed point, limit cycle, quasi-periodic motion, chaotic motion, and periodic chaos, which is similar to multi-scroll behavior. In addition, we have uncovered that the system can collapse due to a boundary crisis via chaos, and even via quasi-periodic motion. The partial collapse as an interesting type of system collapse for certain variables only is found as a quite general phenomenon here. From power systems point of view, the finding of partial collapse for angle variable only indicates that even with the third-order model studied here, rotor-angle stability can only be observed. From nonlinear dynamics point of view, it is also very interesting. All these complicated dynamics are fundamentally different from the behavior for the second-order power system, where the system collapses immediately after the damping is switched from positive to negative. Moreover, two different types of local bifurcations from the equilibrium state, the saddle-node bifurcation and the supercritical Hopf bifurcation, are theoretically analyzed. These analytical results are of great significance to understand the negative-damping-induced stable equilibrium state and to take a fresh look at the negative damping effect and oscillation phenomena in power systems. They are indeed well confirmed by the numerical results, as shown in the phase diagram (Fig 5). In addition, the inertia effects are studied for either positive (γ>0) or negative damping (γ<0) in Figs 4 and 13, respectively. The results show that all complicated dynamics for *M* = 1 can be found for any arbitrarily chosen *M*. However, for positive γ, smaller inertia enlarges the dominant stable fixed point region, and oppositely, for negative γ, larger inertia enlarges the stable fixed point region. Thus, inertia does show different impacts for either positive or negative damping. Further systematical comparison with transient behavior, parameter sensitivity, and dynamical behavior under either noise or intentional disturbance for small or large inertia constants are important.

Finally it is valuable to give further discussions as follows. (1) As the negative damping coefficient is closely related to the synchronous generator angle and the armature resistance, and the negative damping coefficient will affect the stability of a power system, the study in the present work considering both positive and negative damping coefficients is crucial and meaningful. In the third-order model in the paper, we considered the magnetic flux decay and we did not take the controllers into consideration, such as Automatic Voltage Regulator (AVR) and Power System Stabilizer (PSS). The controllers AVR and PSS are just designed to avoid the appearance of negative damping. It has ever been a very hot topic to study these important controllers in power systems [11], e.g., an adaptive power system stabilizer which can cancel the negative damping torque of a synchronous generator was designed [12]. However, it is necessary to investigate these pure negative damping effect in the absence of these classical controllers. (2) Other nonlinear dynamics results here, such as the fixed point, chaos, quasi-periodicity, and different collapses etc, are of great importance to understand the complicated dynamical process in power systems and might help us to design an oscillation damping device. (3) These results considering the voltage dynamics of synchronous generator are also highly relevant to voltage stability and voltage collapse, which are still unsolved hard problems in power systems. (4) When considering only equilibria and their local stability properties, there are quite a few studies, similar to ours, that show the swing equation itself may deliver very different results from higher-order models. See, e.g., several recent papers [7, 8]. (5) Last but not least, for more complicated cases, such as more detailed models for synchronous generator considering damping windings, the interaction of multiple synchronous generators [42–48], different types of stability in power systems including rotor angle stability [49] and voltage stability [50–51], and interplay with delayed power price [52] so on, further theoretic analysis methods have to be developed. It is of interest to study these problems from nonlinear dynamics point of view in the future.

## References

[1] Bergen A R, Vittal V. Power Systems Analysis. Prentive Hall, Upper Saddle River, New Jersey; 1986.

[2] Kundur P. Power System Stability and Control. McGraw-Hill, Inc; 1994.

[3] Paganini F, Lesieutre B C. Generic Properties, One-parameter Deformations, and the BCU Method. IEEE Transactions on Circuits and Systems I: Fundamental Theory and Applications. 2002; 46(6):760–763.

[4] Araspostathis A, Sastry S, Varaiya P. Global Analysis of Swing Dynamics. IEEE Transactions on Circuits and Systems. 1982; Cas-29(10):673–679.

[5] Susuki Y, Mezić I, Hikihara T. Coherent Swing Instability of Power Grids. Journal of Nonlinear Science. 2011; 21(3): 403–439.

[6] Chiang H D. Direct Methods for Stability Analysis of Electric Power Systems: Theoretical Foundation, BCU Methodologies, and Applications. John Wiley & Sons, Inc; 2011.

[7] Caliskan S Y, Tabuada P. Uses and Abuses of the Swing Equation Model. 2015 IEEE 54th Annual Conference on Decision and Control. 2015; 6662–6667.

[8] Monshizadeh P, Persis C D, Monshizadeh N, Schaft A V D. Nonlinear Analysis of an Improved Swing Equation. arXiv preprint arXiv:1603.07440. 2016.

[9] Schaft A V D, Stegink T. Perspectives in Modeling for Control of Power Networks. Annual Reviews in Control. 2016; 41:119–132.

[10] Varaiya P, Wu F F, Chen R L. Direct Methods for Transient Stability Analysis of Power Systems: Recent Results. Proceeding of the IEEE. 2005; 73(12):1703–1715.

[11] Demello F P, Concordia C. Concepts of Synchronous Machine Stability as Affected by Excitation Control. IEEE Transactions on Power Apparatus and Systems. 1969; Pas-88(4):316–329.

[12] Gupta D P S, Narahari N G, Boyd I, Hogg B W. An Adaptive Power-system Stabiliser which Cancels the Negative Damping Torque of a Synchronous Generator. In: Generation, Transmission and Distribution, IEEE Proceeding C. 1985; 132(3):109–117.

[13] Strogatz S H. Nonlinear Dynamics and Chaos: With applications to physics, biology, chemistry, and engineering Perseus Books Publishing, Massachusetts; 1994.

[14] Subbarao D, Singh K K. Hysteresis and Bifurcations in the Classical Model of Generator. IEEE Transactions on Power Systems. 2004; 19(4):1918–1924. 10.1109/TPWRS.2004 836203

[15] Wang X D and Chen Y S. Bifurcation and Singularity Analysis for a Class of Power System. Journal of Vibration and Shock. 2014; 33(4):1–6. (In Chinese)

[16] Wei D Q, Qin Y H. Controlling Chaos in single-machine-infinite bus Power System by Adaptive Passive Method. Chaos-Fractals Theories and Applications. 2011; 8:295–297. 10.1109/IWCFTA.2011.8

[17] Wang X D, Chen Y S, Han G, Song C Q. Nonlinear Dynamic Analysis of a single-machine infinite-bus Power System. Applied Mathematical Modelling. 2015; 39(10):2951–2961. 10.1016/j.apm.2014.11.018

[18] Chen H K, Lin T N, Chen J H. Dynamic Analysis, Controlling Chaos and Chaotification of a SMIB Power System. Chaos, Solitons and Fractals. 2005; 24(5):1307–1315. 10.1016/j.chaos.2004.09.081

[19] Wei D Q, Luo X S. Noise-induced Chaos in single-machine infinite-bus Power System. A Letters Journal Exploring the Frontiers or Physics. 2009; 86(5):50008 10.1209/0295-5075/86/50008

[20] Wei D Q, Zhang B, Qiu D Y, Luo X S. Effect of Noise on Erosion of Safe Basin in Power System. Nonlinear Dynamics. 2010; 61(3):477–482. 10.1007/s1171-010-9663-0

[21] Yuan R X, Ruan Y, Hu P. Nonlinear Excitation Controller Design for Power System: an I&I Approach. J Control Theory Appl. 2012; 10(4):554–558. 10.1007/s11768-012-0168-x

[22] Mahmud M A, Pota H R, Hossain M J. Full-oeder Nonlinear Observer-based Excitation Controller Design for Interconnected Power Systems via Exact Linearization Approach. Electrical Power and Energy Systems. 2012; 41(1):54–62. 10.1016/j.ijepes.2012.03.007

[23] Manjarekar N S, Banavar R N, Ortega R. Application of Interconnection and Damping Assignment to the Stabilization of a Synchronous Generator with a Controllable Series Capacitor. Electrical Power and Energy Systems. 2010; 32(1):63–70. 10.1016/j.ijepes.2009.06.010

[24] Sun Y Z, Li X, Zhao M, Song Y H. New Lyapunov Function for Transient Stability Analysis and Control of Power System with Excitation Control. Electric Power Systems Research. 2001; 57(5):123–131. 10.1016/S0378-7796(01)00085-2

[25] Schmietendorf K, Peinke J, Friedrich R, Kamps O. Self-organized Synchronization and Voltage Stability in Networks of Synchronous Machines. The European Physical Journal Special Topics. 2014: 223(12):2577–2592. 10.1140/epjst/e2014-02209-8

[26] Ji W J, Venkatasubramanian V. Hard-limit Induced Chaos in a Fundamental Power System Model. Electrical Power and Energy Systems. 1996; 18(5):279–295. 10.1016/0142-0615(95)00066-6

[27] Ji W J, Venkatasubramanian V. Hard-limit Induced Chaos in a Single-machine Infinite-bus Power System. Decision and Control. 1995; 4:3465–3470. 10.1109/CDC.1995.479121

[28] Venkatasubramanian V, Ji W J. Coexistence of Four Different Attractors in a Fundamental Power System Model. IEEE Transactions on Circuits and Systems-I: Fundamental Theory and Applications. 1999; 46(3):405–409.

[29] Ma M L, Min F H. Bifurcation Behavior an Coexisting Motions in a Time-delayed Power System. Chin. Phys. B. 2015; 24(3):030501 10.1088/1674-1056/24/3/030501

[30] Abed E H, Varaiya P P. Nonlinear Oscillations in Power Systems. International Journal of Electrical Power and Energy Systems. 1984; 6(1):37–43. 10.1016/0142-0615(84)90034-6

[31] Liwschitz M M. Positive and Negative Damping in Syschronous Machines. Electrical Engineering. 1941; 60(5):210–213. 10.1109/T-AIEE.1941.5058316

[32] Anderson P M, Fouad A A. Power System Control and Stability. Publishing House of Electronics Industry; 2012.

[33] Machowski J, Bialek J W, Bumby J R. Power System Dynamics -Stability and Control. John Wiley and Sons, Ltd; 2008.

[34] Ni Y X, Chen S S, Zhang B L. Dynamic theory and analysis of power system Tsinghua university press; 2002. (In Chinese)

[35] Lu Q, Wang Z H, Han Y D. The Optimal Control of Transmission System. Beijing Science Press; 1982. (In Chinese)

[36] Concordia C, Carter G K. Negative Damping of Electrical Machinery. Transactions of the American Institute of Electrical Engineering. 1941; 60(3):116–118. 10.1109/T-AIEE 1941.5058292

[37] Grebogi C, Ott E, Yorke J A. Crises, Sudden Changes in Chaotic Attractors, and Transient chaos. Physica D 7. 1983; 7(1):181–200. 10.1016/0167-2789(83)90126-4

[38] Wang H O, Abed E H, Hamdan A M A. Bifurcations, Chaos, and Crises in Voltage Collapse of a Model Power System. IEEE Transcations on Circuits and Systems-I: Fundamental Theory and Applications. 1994; 41(3):294–302. 10.1109/81.285684

[39] CHUA L O, Komuro M, Matsumoto T. The Double Scroll Family. IEEE Transactions on Circuits and systems. 1986; 33(11):1072–1118. 10.1109/TCS.1986.1085869

[40] Lv J, Chen G. Generating Multiscroll Chaotic Attractors: Theories, Methods and Applications. International Journal of Bifurcation and Chaos. 2006; 16(4):775–858. 10.1142/S0218127406015179

[41] Govaerts W, Kuznetsov Y A, Witte V D, Dhooge A, Meijer H G E, Mestrom W, et al Matcont and CL-Matcont: Continuation Toolboxes in Matlab. Gent University and Utrecht University; 2011.

[42] Rohden M, Sorge A, Timme M, Witthaut D. Self-organized Synchronization in Decentralized Power Grids. Phys. Rev. Letts. 2012; 109(6):064101 10.1103/PhysRevLett.109.064101 23006269

[43] Motter A E, Myers S A, Anghel M, Nishikawa T. Spontaneous Synchrony in Power-grid Networks. Nature Physics. 2013; 9(3):191–197. 10.1038/nphys2535

[44] Carareto R, Baptista M S, Grebogi C. Natural Synchronization in Power-grids with Anti-correlated Units. Commun Nonlinear Sci Numer Simulat. 2013; 18(4):1035–1046. 10.1016/j.cnsns.2012.08.030

[45] Menck P J, Heitzig J, Kurths J, Schellnhuber H J. How Dead Ends Undermine Power Grid Stability. Nature Communications. 2014; 5:3969 10.1038/ncomms4969 24910217

[46] Simpson-Porco J W, Dorfler F, Bullo F. Voltage Collapse in Complex Power Grids. Nature Communications. 2016; 7:10790 10.1038/ncomms10790 26887284 PMC4759633

[47] Nishikawa T, Motter A E. Comparative analysis of existing models for power-grid synchronization. New Journal of Physics. 2015; 17:015012.

[48] Gajduk A, Todorovski M, Kocarev L. Stability of Power Grids: An overview. Eur. Phys. J. Special Topics. 2014; 223(12):2387–2409.

[49] Dörfler F, Chertkov M, Bullo F. Synchronization in Complex Oscillator Networks and Smart Grids. Proceedings of the National Academy of Sciences. 2013; 110(6): 2005–2010. 10.1073/pnas.1212134110 PMC3568350 23319658

[50] Dobson I, Chiang H D. Towards a Theory of Voltage Collapse in Electric Power Systems. Systems & Control Letters. 1989; 13(3): 253–262.

[51] Dobson I, Zhang J F, Greene S, Engdahl H, Sauer P W. Is strong modal resonance a precursor to power system oscillations? IEEE Transactions on Circuits and Systems I: Fundamental Theory and Applications. 2001; 48(3):340–349.

[52] Quattrociocchi W, Caldarelli G, Scala A. Self-healing Networks: Redundancy and Structure. Plos One.2014; 9(2):e87986 10.1371/journal.pone.0087986 24533065 PMC3922772

